# Antiangiogenic therapy reverses the immunosuppressive breast cancer microenvironment

**DOI:** 10.1186/s40364-021-00312-w

**Published:** 2021-07-22

**Authors:** Wuzhen Chen, Lesang Shen, Jingxin Jiang, Leyi Zhang, Zhigang Zhang, Jun Pan, Chao Ni, Zhigang Chen

**Affiliations:** 1 Department of Breast Surgery (Surgical Oncology), Second Affiliated Hospital, Zhejiang University School of Medicine, 88 Jiefang Road, Hangzhou, 310000 Zhejiang Province China; 2 Key Laboratory of Tumor Microenvironment and Immune Therapy of Zhejiang Province, Hangzhou, China

**Keywords:** Antiangiogenic therapy, Immunotherapy, Breast cancer, Tumor microenvironment (TME)

## Abstract

Tumor angiogenesis induces local hypoxia and recruits immunosuppressive cells, whereas hypoxia subsequently promotes tumor angiogenesis. Immunotherapy efficacy depends on the accumulation and activity of tumor-infiltrating immune cells (TIICs). Antangiogenic therapy could improve local perfusion, relieve tumor microenvironment (TME) hypoxia, and reverse the immunosuppressive state. Combining antiangiogenic therapy with immunotherapy might represent a promising option for the treatment of breast cancer. This article discusses the immunosuppressive characteristics of the breast cancer TME and outlines the interaction between the tumor vasculature and the immune system. Combining antiangiogenic therapy with immunotherapy could interrupt abnormal tumor vasculature-immunosuppression crosstalk, increase effector immune cell infiltration, improve immunotherapy effectiveness, and reduce the risk of immune-related adverse events. In addition, we summarize the preclinical research and ongoing clinical research related to the combination of antiangiogenic therapy with immunotherapy, discuss the underlying mechanisms, and provide a view for future developments. The combination of antiangiogenic therapy and immunotherapy could be a potential therapeutic strategy for treatment of breast cancer to promote tumor vasculature normalization and increase the efficiency of immunotherapy.

## Introduction

The signal imbalance between proangiogenic and antiangiogenic molecules leads to tumor vascular dysfunction [1, 2]. Angiogenesis, which refers to the formation of abnormally immature vessels, often accompanies tumorigenesis. The abnormal structure of the tumor vasculature and restricted blood perfusion prevent immune cells from infiltrating tumors efficiently, which results in an unbalanced and immunosuppressive tumor microenvironment (TME) [2].

Revolutionary changes in cancer treatment have occurred with the continuous development of immune checkpoint blockade (ICB) immunotherapy. However, only 10–30% of breast cancer patients benefit from ICB immunotherapy [3]. Hence, there is a need to explore how to intensify treatment based on immunotherapy to achieve more survival benefits. Although ICB could reactivate dysfunctional or depleted T cells, these reactivated T cells could not infiltrate into the center of solid tumors to exert antitumor effects. Antiangiogenic therapy has been widely studied for a long time [4], and most antiangiogenic agents target vascular endothelial growth factors (VEGFs) and VEGF receptors (VEGFRs) [5]. In preclinical research, antiangiogenic therapy has been shown to reverse abnormal tumor blood perfusion, promote immune cell infiltration and normalize the immune TME [6, 7]. Given this evidence, tumor angiogenesis could interact with the immune TME. Targeting angiogenesis represent a potential option to reverse tumor-associated perfusion abnormalities and the immunosuppressive microenvironment.

In this article, we reviewed the crosstalk between the breast cancer vascular system and the immune microenvironment, discussed the mechanisms by which antiangiogenic therapy reverses the immunosuppressive TME and emphasized the clinical evidence of antiangiogenic therapy plus immunotherapy. We also discussed biomarkers to monitor the response to antiangiogenic agents plus immunotherapy and the challenges in this emerging field.

## Immunosuppressive TME induced by tumor angiogenic factors

Enhanced angiogenesis is the hallmark of cancer. Tumor vasculature is unevenly distributed and chaotic [8]. On the one hand, restricted tumor vascular perfusion blocks the transfer of chemotherapeutic and immunotherapeutic agents to the tumor interior and eliminates infiltrated immunosuppressive cells in the TME. On the other hand, tumor-associated vascular endothelial cells can express programmed death-ligand 1 (PD-L1) and Fas ligand (FasL), selectively inhibit cytotoxic T cells (CTLs), and promote regulatory T cell (Treg) function to enhance the immunosuppressive TME [9, 10]. It has been reported that many tumor angiogenic factors contribute to the immunosuppressive TME, including vascular endothelial growth factors, angiopoietin 2, placental growth factor, and transforming growth factor-β.

### Vascular endothelial growth factors

As a critical factor in tumor angiogenesis, VEGFs could induce an immunosuppressive TME through hypoxia and a low pH [11]. VEGF can bind to VEGFR1 (FLT1) and prohibit dendritic cell (DC) maturation and antigen presentation [12], thus impeding T cell activation and limiting the adaptive antitumor immune response [13]. Increased peripheral VEGF levels are associated with decreased peripheral mature DCs, and anti-VEGF treatment could increase the number of mature DCs and reverse VEGF-mediated immunosuppression [14]. Elevated VEGF-A levels promote CD8^+^ T cell exhaustion by enhancing the expression of PD-1 [15] and contribute to Treg proliferation [16] and myeloid-derived suppressor cell (MDSC) accumulation [17] in the TME. However, Palazon et al. demonstrated that hypoxia and hypoxia-inducible factor-1α (HIF-1α) support the acquisition of an effector phenotype by CD8^+^ T cells, but the activated effector CD8^+^ T cells could produce high levels of VEGF-A [18]. The above results indicate that the regulation mechanism between VEGF and the TME immune status needs be further investigated.

In addition, VEGF-A induces thymocyte selection-associated HMG-bOX (TOX)-mediated depletion of cytotoxic T lymphocytes (CTLs) [19]. TOX is a crucial transcription factor in T cell development and plays a vital role in T cell exhaustion [20]. VEGF-A upregulates the expression of TOX and initiates TOX-mediated reprogramming into an exhausted state in CD8^+^ T cells [19]. Knockout of VEGFR-2 downregulates TOX expression and reactivates tumor-specific exhausted CD8^+^ T cells, indicating the therapeutic potential of targeting the VEGF/VEGFR-2 axis [19].

### Angiopoietin 2

Activated angiopoietin 2 (ANG2) upregulates adhesion molecule expression and recruits bone mesenchymal stem cells (BMSCs), Tregs, and M2-like macrophages expressing the ANG receptor (tyrosine kinase with Ig and EGF homology domains-2, TIE-2) [7, 21]. Additionally, ANG2 suppresses monocyte antitumor function by inhibiting TNF-α secretion [22] and promotes Treg activation and CTL inhibition through interleukin 10 (IL-10) [23].

### Placental growth factor

Placental growth factor (PlGF), as a member of the VEGF family that induces an angiogenic phenotype, directly interacts with VEGFR1 to stimulate tumor angiogenesis and promote macrophage repolarization into the M2-like phenotype, facilitating immune escape [24]. PlGF blockade induces vascular normalization and macrophage phenotypic polarization from an M2-like state to an M1-like state [25].

### Transforming growth factor-β

Transforming growth factor-β (TGF-β) is another important factor that regulates pericyte and endothelial cell proliferation and induces different angiogenic responses according to the balance between activin receptor-like kinase 1 (ALK1) and ALK5 signals. TGF-β/ALK1 signaling promotes endothelial cell proliferation, migration and tube formation by activating SMAD1/5 [26]. In addition, TGF-β inhibits natural killer (NK) cells and T cells to suppress tumor immune surveillance [27].

## TME elements in regulating tumor angiogenesis

**Fig. 1:**
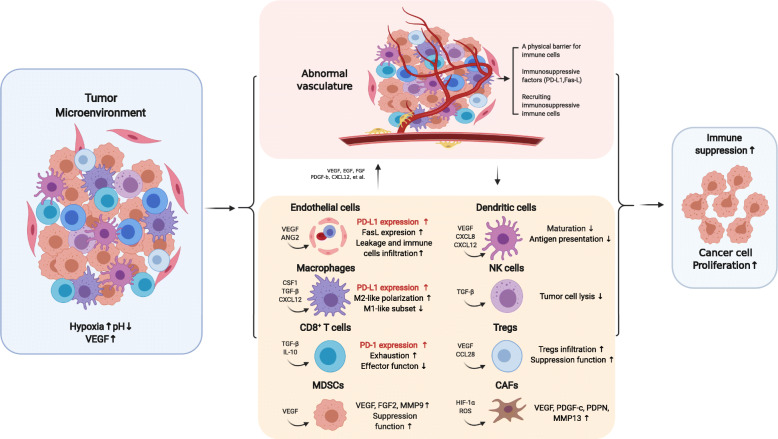
Abnormal tumor vasculature triggers immunosuppression in the tumor microenvironment (created with BioRender.com). Malformed and dysfunctional vascular systems in breast cancer cause perfusion restriction, leading to hypoxia and acidosis in the TME. Tumor vasculature abnormalities promote immunosuppression through multiple mechanisms. VEGF induces tumor angiogenesis, and tumor vascular endothelial cells with PD-L1 and Fas-L expression recruit immunosuppressive cells. CSF1, TGF-β, and CXCL12 polarize TAMs from a protumorigenic M1-like phenotype into an antitumorigenic M2-like phenotype. VEGF, CXCL8, and CXCL12 inhibit DC maturation, resulting in impaired antigen presentation and leading to disrupted T cell activation. TGF-β and IL-10 induce CD8^+^ T cell exhaustion, and TGF-β inhibits NK cell function. Tregs and MDSCs accumulate, activate, and expand in the TME. The CAFs in the TME promote tumor angiogenesis by producing VEGF, PDGF-c, PDPN, and MMP13. The PD-L1 pathway is normally activated as a mechanism to evade the antitumor immune response. Overall, aberrant tumor angiogenesis results in an immunosuppressive TME. ANG2, Angiopoietin 2; CAFs, Cancer-associated fibroblast; CCL28, CC chemokine ligand 28; CXCL8, CXC chemokine ligand 8; CXCL12, CXC chemokine ligand 12; CSF1, Colony-stimulating factor 1; DC, Dendritic cell; Fas-L, FAS antigen ligand; FGF, Fibroblast growth factor; MMP, Matrix metallopeptidase; NK, Natural killer; PD-1, Programmed cell death protein 1; PD-L1, Programmed cell death 1 ligand 1; PDGF, Platelet-derived growth factor; PDPN, Podoplanin; ROS, Reactive oxygen species; TAM, Tumor-associated macrophage; TGF-β, Transforming growth factor beta; TME, Tumor microenvironment; Tregs, Regulatory T cells; VEGF, Vascular endothelial growth factor

Tumor-infiltrating immune cells (TIICs) are deeply involved in the process of tumor angiogenesis, and immunosuppressive cells can promote antiangiogenic therapy resistance by inducing neovascularization in the TME [28, 29] (Fig. 1). Immune cells can secrete proangiogenic or antiangiogenic factors to directly affect the phenotype and function of the tumor vascular endothelium [14, 30, 31] or transform into other immune cell types, which indirectly affects the quantity and quality of tumor blood vessels [32, 33].

### Macrophages

In a clinical trial evaluating immunotherapy in triple-negative breast cancer (TNBC), the expression level of PD-L1 on tumor-associated macrophage (TAMs) was positively correlated with the response to immunotherapy, indicating the vital role of TAMs in the TME [34]. According to differences in functions and secreted cytokines, macrophages are divided into M1-like (antiangiogenic phenotype) and M2-like (proangiogenic phenotype) macrophages [35–37]. M1-like macrophages inhibit angiogenesis and induce vascular maturation by secreting antiangiogenic cytokines (IL-12 and TNF-α) [38, 39]. M1-like macrophages secrete IL-12 to polarize other TAMs into the M1-like phenotype, further reducing the microvascular density through a positive feedback loop [39–41]. However, previous studies have demonstrated that M2-like macrophages are more dominant than M1-like macrophages in the TME [37, 39]. M2-like macrophages promote tumor angiogenesis by producing proangiogenic growth factors (VEGF-A, epidermal growth factor (EGF), and fibroblast growth factor (FGF)), proangiogenic CXC chemokines (CXCL8/IL-8 and CXCL12), and angiogenesis-related factors (TGF-β and TNF-α). These factors enhance the migration and proliferation of endothelial cells and polarize M1-like macrophages into an M2-like phenotype.

The success of antiangiogenic therapy is partly based on macrophage polarization from an M2-like to an M1-like phenotype [42]. Eradication of macrophages with anti-colony stimulating factor 1 (CSF1) antibodies eliminates the benefits of antiangiogenic therapy, suggesting the importance of macrophages in antiangiogenic efficacy [43]. In mouse breast cancer models, elimination of macrophages with clodronate liposomes inhibited tumor angiogenesis and growth [44, 45].

TIE2-expressing macrophages (TEMs) are another unique subtype of TAMs with the capacity to promote tumor angiogenesis [43]. TEMs can bind to ANG2 secreted by endothelial cells or tumor cells and further enhance angiogenesis [46]. Targeted inhibition of TEMs has been indicated to induce tumor vascular normalization and promote tumor regression [47].

### Dendritic cells

DCs, an essential adaptive immune component of the TME, regulate tumor angiogenesis in accordance with the maturation state. Mature DCs suppress tumor angiogenesis by secreting antiangiogenic cytokines (e.g., IL-12 and IL-18) [48, 49]. Moreover, mature DCs release interferon-α (IFN-α) to directly inhibit the proliferation of endothelial cells [50]. In the TME, tumor cells recruit immature DCs from the peripheral blood by releasing multiple cytokines (e.g., VEGF, β-defensin, CXCL12, HGF, and CXCL8), which lack the ability to inhibit tumor angiogenesis [14].

### CD8^+^ CTLs

CD8^+^ CTLs play a key role in inhibiting tumor angiogenesis by secreting IFN-γ [51, 52], which directly inhibits endothelial cell proliferation and tumor vascularization [53] and polarizes M2-like TAMs to an M1-like phenotype [54]. IFN-γ also enhances blood vessel maturation to promote tumor vascular remodeling and inhibit tumor growth by reducing VEGF-A levels and increasing CXCL9, CXCL10 and CXCL11 levels [55, 56].

### Th1, Th2 and Th17 cells

CD4^+^ T helper 1 (Th1) cells help normalize tumor vessels by producing IFN-γ in the TME. In a breast cancer model, Th1 cell activation was shown to improve pericyte coverage, reduce abnormal hyperplasia of tumor vessels, and induce vascular normalization [57, 58]. Th1 cells also inhibit tumor angiogenesis by polarizing M2-like TAMs into M1-like macrophages and inducing DC maturation [8, 59]. Unlike Th1 cells, Th2 cells recruit M2-like macrophages to promote tumor angiogenesis by expressing IL-4, IL-5 and IL-13 [32, 41, 60]. Th17 cells, another subtype of CD4^+^ T helper cells, promote endothelial cell proliferation and tumor angiogenesis by expressing IL-17, a poor prognostic factor in breast cancer [61, 62].

### Tregs

Hypoxic conditions in the TME contribute to Treg proliferation by CCL28 and VEGF overexpression in tumor cells [63, 64]. Tregs secrete VEGF, recruit endothelial cells and promote tumor angiogenesis directly [65]. Furthermore, Tregs indirectly facilitate tumor angiogenesis by inhibiting Th1 cell activation and polarizing TAMs into the M2-like phenotype [35, 41]. Targeted removal of Tregs reduces VEGF levels and inhibits tumor angiogenesis in the TME [66].

### MDSCs

In the TME, MDSCs promote tumor angiogenesis by producing VEGF, FGF2, Bv8, and matrix metalloproteinase (MMP9) [67, 68]. CD11b^+^ Gr1^+^ MDSCs increase intratumor vascular density and reduce tumor necrosis [69, 70]. In addition, MDSCs can be directly involved in tumor angiogenesis by acquiring endothelial cell properties [69, 71]. Reduced MDSCs in the TME are associated with reduced tumor angiogenesis and tumor growth inhibition [72, 73]. Several studies have linked the accumulation of MDSCs to an increase in intratumor VEGF concentrations during disease progression [74]. VEGF stimulates the recruitment of MDSCs, promoting immunosuppression and angiogenesis [75, 76]. MDSCs overcome VEGF inhibition by secreting large amounts of VEGF or interfere with the effects of VEGF-targeted therapy by activating the VEGF-independent proangiogenic signaling pathway [77].

### Cancer-associated fibroblasts (CAFs)

CAFs account for 50–90% of solid tumors and have complex interactions with tumor cells and the extracellular matrix (ECM) [78, 79]. In breast cancer, CAFs secrete stromal cell-derived factor-1 (SDF1), CXC chemokine 12 (CXC12) and VEGF to promote angiogenesis [80–82]. In addition, CAFs secrete podoplanin (PDPN), which can stimulate angiogenesis and lymphangiogenesis by upregulating VEGF-C but not VEGF-A in breast cancer [83–85]. Galectin-1 derived from CAFs accelerates angiogenesis and promotes tumor invasion by enhancing VEGF expression in tumor cells and VEGFR2 phosphorylation in epithelial cells (ECs) [86–89]. In the hypoxic TME of breast cancer, G-protein-coupled estrogen receptor (GPER), HIF-1α and reactive oxygen species (ROS) are involved in CAF activation and VEGF expression upregulation to promote hypoxia-dependent tumor angiogenesis [90, 91]. CAFs can release ECM-bound VEGF by secreting MMP-13 [92]. CAFs also promote tumor angiogenesis in a VEGF-independent manner. In chemotherapy-resistant tumors, increased expression of platelet-derived growth factor-c (PDGF-c) by CAFs contributes to tumor angiogenesis during anti-VEGF therapy [93].

## Antiangiogenic therapy reverses the immunosuppressive TME

### Antiangiogenic therapy promotes TIIC accumulation

Antiangiogenic therapy could normalize tumor vessels by pruning immature vessels [94], provides paths for immune cell infiltration and recruits effector TIICs [95]. Firstly, antiangiogenic treatment has been identified to induce DC maturation and promote antigen presentation [18, 96]. Secondly, it upregulates the expression of adhesion molecules (e.g., intercellular adhesion molecule-1 (ICAM1) and vascular cell adhesion molecule-1 (VCAM1)) during the vascular normalization window and helps T cells cross the endothelial barrier and promote CD8^+^ T cell accumulation [7, 97]. Thirdly, it transforms M2-like TAMs into the M1 phenotype [98]. Meanwhile, antiangiogenic therapy reduces the levels of immunosuppressive TIICs including Tregs and MDSCs in peripheral blood, accompanied by an improvement in the Th1 cell response [99]. High endothelial venules (HEVs) are specialized vascular units organized in tertiary lymphoid structures that recruit immature T cells and help immature T cells differentiate into CTLs [100]. Endothelial cells in HEVs support the homing and migration of effector immune cells into the tumor via ICAM1 [101]. HEVs are remodeled by VEGF-D in tumor tissues, expand and lose their typical morphology and lymphocyte transport-related molecular features (loss of CCL21 expression) [102–104]. Antiangiogenic therapy helps restore the typical morphology of HEVs and promotes lymphatic drainage [100]. Antiangiogenic therapy also upregulates PD-L1 expression on endothelial cells and tumor cells in mouse breast cancer models [9, 101], which sensitizes the tumor cells to anti-PD-1 therapy [7].

### High dose or low dose?

High-dose or long-term antiangiogenic therapy causes large-scale vascular pruning in vitro, which aggravates hypoxia or acidosis in the TME and promotes immunosuppression, suggesting that the optimal doses of antiangiogenic drugs need to be further explored [105, 106]. When excessive vessels are overpruned or alternative angiogenic pathways are activated, the window of vascular normalization could close [107]. In a study of a hepatocellular carcinoma model, blocking VEGF signaling with high-dose sorafenib aggravated TME hypoxia and promoted the recruitment of immunosuppressive Tregs and M2-like macrophages [7]. In addition, excessive antiangiogenic therapy could produce a hypoxic environment that favors cancer stem cell survival [108]. In contrast, lower doses of antiangiogenic agents are likely to maintain long-term vascular normalization [2]. The antitumor activities of TIICs could be improved by normalizing vessels, reducing tumor hypoxia, and restoring the physiological pH [109]. Hence, to realize the full potential of antiangiogenic therapy, the antiangiogenic regimen and dose need to be adjusted according to the baseline level of the microvascular density (MVD) and pretreatment level of circulating VEGF [110, 111].

### Mono-blockade or dual-blockade?

Antiangiogenic therapy could create a vascular normalization window and improve the delivery of therapeutic drugs and effector immune cells [112]. The process of vascular normalization is short and reversible, and the normalization window is typically short (from weeks to months), depending on the type and dose of antiangiogenic agent [7, 113]. Tumors can evade antiangiogenic therapy through upregulation of alternative angiogenic pathways (e.g., ANG2/TIE2 signaling) [42, 43]. In melanoma, peripheral ANG2 levels represent an effective predictor of ICB immunotherapy response with increased ANG2 levels indicating no response to ICB immunotherapy [114]. Compared with anti-VEGF or anti-ANG2 monotherapy, dual blockade of VEGF and ANG2 relieves TME immunosuppression [43] and prolongs the vascular normalization window [115] and overall survival (OS) in preclinical studies [9, 116, 117]. Furthermore, the dual blockade of VEGF and ANG2 promotes the accumulation of CD4^+^ and CD8^+^ T cells and increases IFN-γ levels in the TME [9, 117]. However, it is crucial to select the proper doses for dual antiangiogenic therapy to avoid excessive vascular pruning and increase the delivery of chemotherapeutic drugs [118]. Therefore, targeting VEGF and ANG2 simultaneously improves the efficacy of antiangiogenic therapy and promotes the restoration of antitumor immunity in the TME.

### Immunotherapy promotes vascular normalization

Immunotherapy normalizes vessels in various tumor models, and vascular normalization is attributed to the accumulation and increased antitumor activities of Th1 cells in breast cancer [7, 100]. In CD4+ T cell-deficient mouse mammary tumor models, pericyte coverage of blood vessels was reduced, and tumor tissue hypoxia was increased, suggesting that CD4+ T cell deficiency led to vascular abnormalities [57]. ICB activates CD4+ and CD8+ T cells in the TME, remodels the tumor vasculature, and indirectly enhances their antitumor activity [30]. The accumulation and reactivation of effector T cells in the TME subsequently helps long-term tumor control indirectly [119]. In addition, tumor tissue hypoxia leads to an increase in Tregs [66, 120, 121]. Tregs promote tumor angiogenesis, and the depletion of Tregs activates CD8+ T cells and promotes vascular normalization [66, 122]. Understanding the vascular normalization function of ICB immunotherapy is helpful to optimize the administration sequence of ICB and antiangiogenic agents to expand the window of normalization and extend the survival time of breast cancer patients [123].

As a new target of immunotherapy, stimulator of interferon gene (STING) has been associated with tumor vascular system regulation and has shown a synergistic effect with anti-VEGF2 antibodies and ICB [52]. Activated STING signaling inhibits tumor angiogenesis and induces vascular normalization through activation of type I IFN signaling [14]. Intriguingly, CD8^+^ CTLs are implicated in vascular remodeling triggered by STING signaling. STING agonists and anti-VEGF2 antibodies synergistically promote vascular normalization and prolong antitumor immunity [14]. It is worth noting that STING-based immunotherapy is effective in overcoming antiangiogenic therapy or ICB monotherapy resistance [52]. Thus, the tumor vascular normalization effects of immunotherapy provide a new understanding of tumor vascular remodeling and immune reprogramming. Nevertheless, the conditions required for immunotherapy-induced vascular normalization, the duration of the response, and the distinction from antiangiogenic therapy-mediated vascular normalization remain unclear [30, 119].

### The influence of tumor MHC-I expression

To avoid recognition by CD8+ T cells, tumor cells have adopted an immune evasion strategy of loss of MHC I expression [124–127]. The majority of early-stage tumors are MHC-I positive [128]. Tumor-resistant CD8+ T cells exert evolutionary selection pressure on tumor MHC-I-positive cells, resulting in defective or negative MHC-I expression in tumors [129, 130]. A study showed that deletion of MHC-I expression was associated with resistance to ICB immunotherapy [131]. The low MHC-I expression hides tumor mutation neoantigens, which explains why some tumors (even with high TMB) do not respond to ICB [132]. Furthermore, during immunotherapy (interferon-α/autologous vaccination), metastases with high MHC-I expression were regressive, whereas metastases with low MHC-I expression were progressive [130].

Antiangiogenic therapy potentially represents an optional approach to overcome MHC-I low expression tumors with immunotherapy resistance. Wallin et al. demonstrated that the combination of bevacizumab and atezumab for metastatic renal cell carcinoma promoted antigen-specific T cell migration and elevated intratumor MHC-I, Th1 and T effector cell markers and chemokines (most notably CX3CL1) [133]. In addition, antiangiogenic therapy normalizes tumor vasculature by reducing microvessel density and improving pericyte coverage, avoiding the influence of tumor MHC-I expression. Therefore, it is necessary to design prospective clinical trials to explore the antitumor effects of antiangiogenic therapy on tumors with low MHC-I expression.

## Antiangiogenic plus immunotherapy promotes TME normalization

### Antiangiogenic therapy and immunotherapy in different molecular subtypes of breast cancer

Previous literature suggests that microvascular density (MVD) levels are higher in TNBC than in other breast cancer subtypes [134] and that angiogenesis in TNBC is increased [135, 136]. In neoadjuvant chemotherapy, the addition of bevacizumab improved the pCR rate in TNBC patients [137–139]. In metastatic TNBC patients, chemotherapy combined with bevacizumab improved progression-free survival (PFS) [140–143]. However, adding bevacizumab to chemotherapy resulted in an increased incidence of adverse events and did not improve overall survival in patients with metastatic TNBC [144]. In adjuvant chemotherapy, chemotherapy combined with bevacizumab did not improve invasive disease-free survival (iDFS) or OS in TNBC patients [145]. In summary, the addition of anti-vascular therapy to TNBC treatment may improve the clinical response, but there is no clinical evidence for improvement in OS. The clinical benefit of antiangiogenic monotherapy in TNBC remains controversial, and the therapeutic effects of multitarget tyrosine kinase inhibitors (TKIs) need to be further evaluated [146]. In the luminal or HER2-enriched subtype of breast cancer, anti-HER2 trastuzumab and metronomic chemotherapy could also induce vascular normalization by upregulating the expression of thrombospondin-1 (THBS1) in breast cancer [147, 148]. Similarly, cyclin-dependent kinase 4 (CDK4) and CDK6 inhibitors could enhance the efficacy of immunotherapy through vascular normalization [149].

Recently, most studies of ICB immunotherapy in breast cancer have focused on TNBC. Based on the positive results of the IMpassion130 trial, atezolizumab combined with paclitaxel was approved by the FDA for first-line treatment of inoperable locally advanced or metastatic PD-L1^+^ TNBC [3]. The KEYNOTE-355 trial was a randomized, double-blind, phase III trial that evaluated pembrolizumab plus chemotherapy (albumin-bound paclitaxel, paclitaxel or gemcitabine/carboplatin) as a first-line treatment for locally advanced or metastatic TNBC. The primary endpoint of progression-free survival (PFS) was achieved in PD-L1^+^ patients with a combined positive score (CPS) ≥ 10 [150]. Compared with those for immunotherapy in advanced breast cancer, the results for immunotherapy in the neoadjuvant setting are more encouraging. In the phase II I-SPY2 trial, the pathological complete response (pCR) rate was increased by 38% (22 to 60%) by the addition of pembrolizumab. This result might be attributed to the immunostimulatory effect of anthracyclines, which boosts intratumoral immunity and antigen presentation [151]. The phase II GeparNeuvo study showed an increased pCR rate in the durvalumab-pretreated group (61.0% vs. 41.4%) [152, 153]. In the phase III KEYNOTE-522 trial, the addition of pembrolizumab to neoadjuvant chemotherapy increased the pCR rate of patients with early TNBC (64.8% vs. 51.2%), and pembrolizumab immunotherapy extended event-free survival (EFS) by 18 months (HR 0.63; 95% CI 0.43–0.93) [154]. Immunotherapy is more effective in the neoadjuvant phase, which may be due to better PD-L1 positivity rates being found in neoadjuvant patients with better baseline levels and physical status compared with advanced breast cancer patients.

### The exploration of antiangiogenic plus immunotherapy

**Fig. 2:**
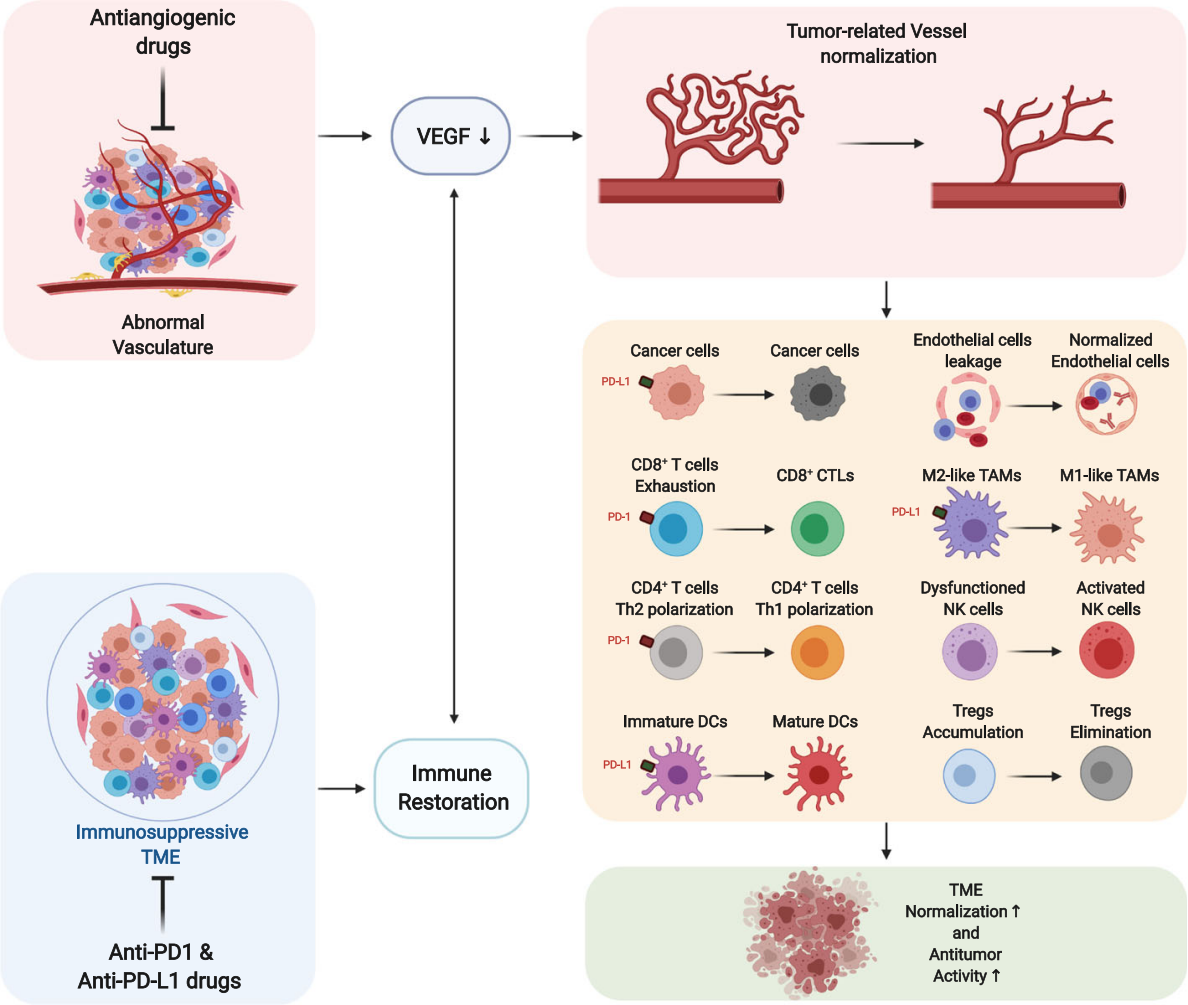
Antiangiogenic therapy combined with immunotherapy improves the tumor immune microenvironment (created with BioRender.com). In breast cancer, antiangiogenic therapy (bevacizumab or VEGFR-TKI) induces tumor vascular normalization, improves blood perfusion, and promotes immune cell recruitment and dendritic cell (DC) maturation. The immunosuppressive state is further relieved using immune checkpoint inhibitors (anti-PD-1/PD-L1 monoclonal antibodies, mAbs). After A + I combination therapy, the immunosuppressive microenvironment is transformed into an immune-supporting microenvironment with increased numbers of M1-like macrophages, mature DCs, CD8+ CTLs, Th1 CD4+ T cells, and activated NK cells and decreased numbers of Tregs, thus effectively exerting an antitumor effect. CTL, Cytotoxic T cell; ANG2, Angiopoietin 2; DC, Dendritic cell; Fas-L, FAS antigen ligand; FGF, Fibroblast growth factor; MMP, Matrix metallopeptidase; NK, Natural killer; PD-1, Programmed cell death protein 1; PD-L1, Programmed cell death 1 ligand 1; TAM, Tumor-associated macrophage; TME, Tumor microenvironment; Tregs, Regulatory T cells; VEGF, Vascular endothelial growth factor

Studies have been designed to study the feasibility and function of antiangiogenic plus immunotherapy (A + I) combined therapy in TME normalization (Fig. 2). In unresectable hepatocellular carcinoma, compared with sorafenib, atezolizumab in combination with bevacizumab reduced mortality (HR 0.58; 95% CI 0.42–0.79; *P* < 0.001) and improved overall survival (67.2% vs 54.6%) [155]. In metastatic non-squamous non-small cell lung cancer, the addition of atezolizumab to bevacizumab combination chemotherapy significantly improved overall survival (19.2 vs. 14.7 months; HR 0.78; 95% CI 0.64 to 0.96; *P* = 0.02) [156]. In addition, PD-L1 expression in tumor tissue could not serve as a biomarker to predict the response to A + I combination therapy [156]. In advanced renal cell carcinoma, compared with sunitinib, treatment with axitinib combined with pembrolizumab [157] or with alvumab [158] increased progression-free survival. In advanced endometrial cancer, lenvatinib plus pembrolizumab showed good antitumor activity [159]. In breast cancer, immunotherapy was administered to patients who received first-line bevacizumab to determine whether ICB could restore sensitivity to antiangiogenic agents. A recently published phase Ib study recruited patients with metastatic HER2-negative breast cancer who had progressed after at least 6 weeks of first-line treatment with bevacizumab, and these patients were treated with durvalumab plus bevacizumab [160, 161]. Interestingly, the patients whose disease remained stable at the first evaluation (2 months) showed a 1.2- to 3.5-fold increase in CD8^+^ effector memory T cell levels in the peripheral blood, but no such change was observed in patients with disease progression [160, 161]. The benefits of antiangiogenic drugs are time and dose dependent, and determining the window of normalization of tumors is challenging [119]. Clinical trials have shown that the combination of low-dose regorafenib with nivolumab is superior to high-dose therapy in advanced gastric or colorectal cancer [162], suggesting that the administration of immunotherapy with antiangiogenic therapy protects against excessive pruning of blood vessels [163]. Further studies are needed to investigate the clinical benefits of A + I combination therapy in breast cancer, especially for TNBC [164].

### Antiangiogenic therapy reduces the adverse events of ICB immunotherapy

Most adverse events related to immunotherapy are linked with a hyperactive immune response, such as T cell-mediated autoimmune inflammation and immune homeostasis disorder, which may lead to immune-related damage to normal tissues, including the gastrointestinal tract, skin and liver. These adverse events could be alleviated by interrupting or reducing the dose of ICB immunotherapy [165]. Considering that vascular normalization could improve the delivery of therapeutic drugs, the proposed combination strategy may require lower doses of ICB to enhance immune responses while reducing the risk of adverse effects [7]. Notably, ICB immunotherapy increases the risk of brain edema, occasionally leading to death [166]. In contrast, antiangiogenic drugs could reduce brain edema, providing theoretical support for combined low-dose antiangiogenic therapy and immunotherapy in the treatment of brain metastases [167]. Based on current clinical data, some ICB agents (e.g., SHR-1210) could cause reactive capillary hemangioma [168], whereas antiangiogenic therapy could suppress hemangioma and reduce anti-PD-1-related adverse effects [169]. For antiangiogenic therapy, the common adverse effects include hypertension, hemorrhage, thrombosis, and proteinuria. Breast cancer is a type of tumor with connective tissue hyperplasia, and increases in the levels of extracellular matrix molecules (including type I collagen and hyaluronan) compress vessels and lead to hypoxic conditions. Angiotensin receptor blockers (ARBs) normalize the matrix and decompress vessels, reducing the adverse effects of antiangiogenic therapy [170]. In addition, ARBs activate both the innate and adaptive immune systems [171] and might improve the effects of A + I combination therapy [172, 173].

### Serum-based biomarkers

A + I combination therapy improves the tumor tissue perfusion status and activates the local immune response; therefore, it is of clinical importance to identify relevant biomarkers reflecting the vascular-immune status in the TME. Serum biomarkers, which have previously been used to monitor response to antiangiogenic therapies [174, 175], could be explored for predicting response to antiangiogenic combination immunotherapy. Serum ANG2, a key factor in vascular maturation [176], was negatively associated with clinical response rates and overall survival to anti-CTLA4 immunotherapy in melanoma [114]. In tumor vaccine-treated NSCLC patients, ANG2 and VEGF-A serum levels could be predictive factors for long-term remission and survival [30]. Together, these findings support a correlation between tumor vascular remodeling and antitumor immune response generation, suggesting a potential role for the use of vascular-related biomarkers to predict clinical response to anti-vascular combination immunotherapy. However, serum-based biomarkers are disturbed by the host physical status, and whether they truly reflect the status of the TME requires further investigation. In addition, whether novel serum biomarkers, such as exosomes, circulating tumor DNA (ctDNA), serum RNA, immune cell subpopulation counts and lactate dehydrogenase (LDH) levels, have clinical predictive significance deserves further exploration.

### Tissue-based biomarker

The main limitation of tissue-based biomarkers is the need for repeat biopsies. In metastatic breast cancer, tumor tissue PD-L1 expression and tumor mutational burden (TMB) could be predictors of immunotherapy efficacy [3, 7]. In contrast, for breast cancer neoadjuvant treatment, predictors of immunotherapy efficacy still need to be further explored [153]. Mpekris et al. investigated the complex interactions among tumor cells, immune cells (M1/M2-like TAMs, NK cells, CD4+ / CD8+ T cells, and Tregs), and endothelial cells and developed a mathematical model for tumor tissue perfusion assessment and immunotherapy efficacy prediction [119]. The model was designed considering the levels of proangiogenic molecules (e.g., ANG1, ANG2, PDGF-b, VEGF and CXCL12) in the TME and the vascular normalization effect of CD4+ and CD8+ T cells [119]. The model predictions exhibit good correlation with the preclinical results, which need further validation in prospective clinical studies.

The efficacy of immunotherapy depends on tumor perfusion, and any approach to improve perfusion could simultaneously enhance immunotherapy. The incidence of HEVs in tumors might also predict the effect of A + I combination therapy [146]. The formation of HEVs has been demonstrated to improve the effects of ICB immunotherapy [177]. In breast cancer models, HEV formation was mediated by lymphotoxin-β receptor (LT-βR) signal transduction. Treatment with an agonistic LT-βR antibody induced HEV development and increased CTL activation, further enhancing the efficacy of antiangiogenic therapy [101].

In addition, functional measurements of vascular changes by noninvasive measures, such as dynamic contrast enhanced (DCE) MRI [178], dynamic optical breast imaging (DOBI) [179], and shear-wave elastography (SWE) [180], might be helpful in A + I combination therapy. These functional measurements could provide important information about the TME status and allow dynamic monitoring during treatment. The biomarkers and cells described above in the breast cancer TME are summarized in Table 1.

**Table 1:** Biomarkers and cells in the breast cancer microenvironment

**TME elements**	**Function**
M1-like TAMs	Suppress angiogenesis (IL-12 and TNF-α); Induce vascular maturation
M2-like TAMs	Promote angiogenesis (VEGF-A, EGF and FGF); Enhance ECs migration and proliferation; Polarize M1-like into M2-like TAMs
Mature dendritic cells	Suppress angiogenesis (IL-12 and IL-18); inhibit EC proliferation
Immature dendritic cells	Lack the ability to inhibit angiogenesis
CD8+ CTLs	Suppress angiogenesis (IFN-γ)
Th1 cells	Suppress angiogenesis (IFN-γ); improve pericyte coverage
Th2 cells	Promote angiogenesis (IL-4, IL-5 and IL-13); recruit M2-like TAMs
Th17 cells	Promote angiogenesis (IL-17); promote ECs proliferation
Tregs	Promote angiogenesis (VEGF); inhibit Th1 cell activation; promote M2-like TAMs
MDSCs	Promote angiogenesis (VEGF, FGF2, Bv8 and MMP9); acquire endothelial cell properties
CAFs	Promote angiogenesis (VEGF, CXC12, SDF1 and PDGF-c); enhance VEGF expression (PDPN and LGALS1); release ECM-bound VEGF
**Serum-based biomarkers**	**Function**
ANG2 and VEGF-A	Key factor of angiogenesis; predict long-term remission and survival
**Tissue-based biomarkers**	**Function**
HEVs	Specialized vascular units organized in tertiary lymphoid structures; help immature T cells differentiate into CTLs; the formation of HEVs indicates the improvement of ICB immunotherapy efficacy
TME model	Investigate the complex interactions between tumor cells, immune cells (M1/M2-like TAMs, NK cells, CD4^+^ / CD8^+^ T cells, and Tregs), and endothelial cells; assess tissue perfusion and predict immunotherapy efficacy
Noninvasive measures (DCE-MRI, DOBI, SWE)	Measure vascular changes and provide information on TME status

*ANG2* Angiopoietin 2, *CAF* Cancer-associated fibroblast, *CTL* Cytotoxic T lymphocyte, *DCE* Dynamic contrast enhanced, *DOBI* Dynamic optical breast imaging, *EC* Epithelial cell, *EGF* Epidermal growth factor, *FGF* Fibroblast growth factor, *HEV* High endothelial venules, *ICB* Immune checkpoint blockade, *MDSC* Myeloid-derived suppressor cell, *MMP* Matrix metallopeptidase, *SWE* Shear-wave elastography, *TAM* Tumor-associated macrophage, *TME* Tumor microenvironment, *VEGF* Vascular endothelial growth factor

### Prospects of antiangiogenic therapies in breast cancer

To verify clinical efficacy, several clinical trials using different combinations of antiangiogenic therapies and immunotherapies have been conducted (Table 2). According to the Clinicaltrials.gov registry, most ongoing clinical trials (9/11) focus on patients with advanced breast cancer, whereas 2/11 trials focus on the use of antiangiogenic therapy and immunotherapy in the neoadjuvant phase.

**Table 2:** Currently enrolled clinical studies of antiangiogenic immunotherapy combinations for breast cancer (data source: clinicalTrials.gov, Oct 2020)

No	Title	Status	Conditions	Interventions	Locations
1	A Study to Describe the Diagnosis, Anti-Cancer Treatment and Clinical Outcome in Patients with Newly Diagnosed Breast Cancer in Latin America	Recruiting	Breast Cancer	Drug: Bevacizumab, Drug: Trastuzumab, Drug: Ado-trastuzumab emtansine, Drug: Pertuzumab, Drug: Atezolizumab, Drug: Capecitabine	Instituto Alexander Fleming, Buenos Aires, Argentina, and more
2	A Multi-cohort Phase II Study of HER2-positive and Triple- negative Breast Cancer Brain Metastases.	Not yet recruiting	Breast Cancer	Drug: Pyrotinib, Drug: Temozolomide Injection, Drug: SHR-1316 (PD-L1), Drug: Bevacizumab, Drug: Cisplatin/Carboplatin	Fudan University Shanghai Cancer Center
3	Pre-operative Immunotherapy Combination Strategies in Breast Cancer	Recruiting	Breast Cancer, Estrogen Receptor-positive Breast Cancer	Drug: Atezolizumab, Drug: Cobimetinib, Drug: Ipatasertib, Drug: Bevacizumab	Barts Health NHS Trust, London, United Kingdom
4	Safety and Efficacy of Toripalimab in HER2- Metastatic Breast Cancer Patients Treated with Metronomic Vinorelbine	Recruiting	Metastatic Breast Cancer	Drug: Vinorelbine 40 mg, Biological: Toripalimab 240 mg (PD-1), Biological: Bevacizumab 15 mg/kg, Drug: Cyclophosphamide 50 mg, Drug: Capecitabine 500 Mg Oral Tablet, Drug: Cisplatin, Radiation: Hypofractionated radiotherapy	Cancer Hospital, Chinese Academy of Medical Sciences, Beijing, Beijing, China, and more
5	SAFIR02_Breast - Efficacy of Genome Analysis as a Therapeutic Decision Tool for Patients with Metastatic Breast Cancer	Active, not recruiting	Metastatic Breast Cancer	Drug: AZD2014, Drug: AZD4547, Drug: AZD5363, Drug: AZD8931, Drug: MEDI4736, Drug: Anthracyclines, Drug: Taxanes, and 12 more	Institut de Couldcérologie de l’Ouest/Paul Papin, Angers, France, and more
6	A Study Evaluating the Efficacy and Safety of Multiple Immunotherapy-Based Treatment Combinations in Patients with Metastatic or Inoperable Locally Advanced Triple- Negative Breast Cancer	Recruiting	Triple-negative Breast Cancer	Drug: Capecitabine, Drug: Atezolizumab, Drug: Ipatasertib, Drug: SGN-LIV1A, Drug: Bevacizumab, Drug: Chemotherapy (Gemcitabine + Carboplatin or Eribulin), Drug: Selicrelumab, Drug: Tocilizumab, Drug: Nab-Paclitaxel, Drug: Sacituzumab Govitecould	University of California San Diego Medical Center; Moores Cancer Center, La Jolla, California, United States, and more
7	QUILT-3.067: NANT Triple Negative Breast Cancer (TNBC) Vaccine: Molecularly Informed Integrated Immunotherapy in Subjects with TNBC Who Have Progressed on or After Standard-of-care Therapy.	Active, not recruiting	Triple-negative Breast Cancer	Drug: Aldoxorubicin HCl, Biological: N-803, Biological: ETBX-011, Biological: ETBX-051, Biological: ETBX-061, Biological: GI-4000, Biological: GI-6207, Biological: GI-6301, Biological: haNK for Infusion, Biological: avelumab, and 8 more	Chan Soon-Shiong Institute for Medicine, El Segundo, California, United States
8	A Study of Multiple Immunotherapy-Based Treatment Combinations in Hormone Receptor (HR)-Positive Human Epidermal Growth Factor Receptor 2 (HER2)-Negative Breast Cancer	Recruiting	Breast Neoplasms	Drug: Atezolizumab, Drug: Bevacizumab, Drug: Entinostat, Drug: Exemestane, Drug: Fulvestrant, Drug: Ipatasertib, Drug: Tamoxifen, Drug: Abemaciclib	University of Alabama at Birmingham, Birmingham, Alabama, United States, and more
9	Evaluation of IPI-549 Combined with Front-line Treatments in Pts. With Triple-Negative Breast Cancer or Renal Cell Carcinoma (MARIO-3)	Recruiting	Breast Cancer, Renal Cell Carcinoma	Drug: IPI-549, Drug: Atezolizumab, Drug: nab-paclitaxel, Drug: Bevacizumab	Ironwood Cancer and Research Center, Chandler, Arizona, United States, and more
10	I-SPY 2 TRIAL: Neoadjuvant and Personalized Adaptive Novel Agents to Treat Breast Cancer	Recruiting	Breast Neoplasms, Breast Cancer, Breast Tumors, Angiosarcoma	Drug: Standard Therapy, Drug: AMG 386 with or without Trastuzumab, Drug: AMG 479 (Ganitumab) plus Metformin, Drug: MK-2206 with or without Trastuzumab, Drug: AMG 386 and Trastuzumab, Drug: T-DM1 and Pertuzumab, Drug: Pertuzumab and Trastuzumab, Drug: Ganetespib, Drug: ABT-888, Drug: Neratinib, and 11 more	University of Alabama at Birmingham, Birmingham, Alabama, United States, and more
11	A Phase I/II Study of MEDI4736 in Combination with Olaparib in Patients With Advanced Solid Tumors.	Active, not recruiting	Ovarian, Breast, SCLC, Gastric Cancers	Drug: Olaparib, Drug: MEDI4736, Drug: Bevacizumab	Research Site, Newnan, Georgia, United States, and more

Due to the narrow window of antiangiogenic therapy and the low positivity rate of PD-L1 in patients with advanced breast cancer, the combined use of antiangiogenic therapy and immunotherapy in early-stage breast cancer may produce better clinical benefits. Combining antiangiogenic therapy and immunotherapy may be more promising in the neoadjuvant setting, whereas the timing of antiangiogenic therapy and surgery should also be considered. In addition, current combined antiangiogenic treatments mainly focus on monoclonal antibodies (e.g., bevacizumab). Investigating the effects of multitarget TKIs in combination with immunotherapy is also needed in future clinical trials.

## Conclusion

Antiangiogenic therapies normalize tumor vasculature, improve tissue perfusion, and promote the aggregation of TIICs in the TME. This mechanism forms the basis for combining antiangiogenic therapy with immunotherapy. With the advancement of preclinical and clinical studies, persuasive evidence supports that A + I combination therapy could reverse the immunosuppressive TME and yield overall prognostic improvement for breast cancer patients. However, A + I combination therapy has complex biological effects that might increase the risk of hemorrhage, hypertension, and immune-related adverse effects. A considerable number of clinical trials are currently underway to determine whether A + I combination therapy promotes TME normalization and improves breast cancer survival, especially for TNBC. Given the high cost and side effects of A + I combination therapy, further investigation on relevant biomarkers for A + I combination therapy, especially serum-based biomarkers and tissue-based noninvasive measurements for TME status detection, is needed.

## Data Availability

Not applicable.

## Abbreviations

ALK: Activin receptor-like kinase
ANG2: Angiopoietin 2
ARB: Angiotensin receptor blocker
BMSC: Bone mesenchymal stem cell
CAFs: Cancer-associated fibroblast
CCL28: CC chemokine ligand 28
CDK: Cyclin-dependent kinase
CPS: Combined positive score
CSF1: Colony stimulating factor 1
CTL: Cytotoxic T lymphocyte
CXC12: CXC chemokine 12
CXCL8: CXC chemokine ligand 8
CXCL12: CXC chemokine ligand 12
DC: Dendritic cell
DCE: Dynamic contrast enhanced
DOBI: Dynamic optical breast imaging
EC: Epithelial cell
ECM: Extracellular matrix
EFS: Event-free survival
EGF: Epidermal growth factor
FasL: Fas ligand
FGF: Fibroblast growth factor
FLT1: Fms Related Receptor Tyrosine Kinase 1
GPER: G-protein-coupled estrogen receptor
HEV: High endothelial venule
HIF-1α: Hypoxia-inducible factor-1α
HR: Hazard ratio
ICAM1: Intercellular adhesion molecule-1
ICB: Immune checkpoint blockade
iDFS: Invasive disease-free survival
IL: Interleukin
LGALS1: Galectin-1
LT-βR: Lymphotoxin-β receptor
mAb: Monoclonal antibody
MDSC: Myeloid-derived suppressor cell
MMP: Matrix metallopeptidase
MVD: Microvascular density
NK: Natural killer
OS: Overall survival
pCR: Pathological complete response
PDGF: Platelet-derived growth factor
PDPN: Podoplanin
PFS: Progression-free survival
PLGF: Placental growth factor
ROS: Reactive oxygen species
SDF1: Stromal cell-derived factor-1
STING: Stimulator of interferon gene
SWE: Shear-wave elastography
TAM: Tumor-associated macrophage
TEM: TIE2-expressing macrophage
TGF-β: Transforming growth factor-β
THBS1: Thrombospondin-1
TIIC: Tumor-infiltrating immune cell
TKI: Tyrosine kinase inhibitor
TMB: Tumor mutational burden
TME: Tumor microenvironment
TNBC: Triple-negative breast cancer
TOX: Thymocyte selection-associated HMG-box
Tregs: T regulatory cells
VCAM1: Vascular cell adhesion molecule-1
VEGF: Vascular endothelial growth factor
VEGFR: Vascular endothelial growth factor receptor
